# The cost and cost-effectiveness of rapid testing strategies for yaws diagnosis and surveillance

**DOI:** 10.1371/journal.pntd.0005985

**Published:** 2017-10-26

**Authors:** Christopher Fitzpatrick, Kingsley Asiedu, Anita Sands, Tita Gonzalez Pena, Michael Marks, Oriol Mitja, Filip Meheus, Patrick Van der Stuyft

**Affiliations:** 1 Department of Control of Neglected Tropical Diseases, World Health Organization, Geneva, Switzerland; 2 Department of Chemistry, University of North Carolina at Chapel Hill, Chapel Hill, United States of America; 3 Clinical Research Department, Faculty of Infectious and Tropical Diseases, London School of Hygiene and Tropical Medicine, London, United Kingdom; 4 Barcelona Institute for Global Health, Hospital Clinic -University of Barcelona, Barcelona, Spain; 5 Unit of Epidemiology and Control of Neglected Tropical Diseases, Institute of Tropical Medicine, Antwerp, Belgium; 6 Prevention and Implementation Group, International Agency for Research on Cancer, Lyon, France; 7 Unit of General Epidemiology and Disease Control, Institute of Tropical Medicine, Antwerp, Belgium; 8 Department of Public Health, Ghent University, Ghent, Belgium; University of Connecticut Health Center, UNITED STATES

## Abstract

**Background:**

Yaws is a non-venereal treponemal infection caused by *Treponema pallidum* subspecies *pertenue*. The disease is targeted by WHO for eradication by 2020. Rapid diagnostic tests (RDTs) are envisaged for confirmation of clinical cases during treatment campaigns and for certification of the interruption of transmission. Yaws testing requires both treponemal (trep) and non-treponemal (non-trep) assays for diagnosis of current infection. We evaluate a sequential testing strategy (using a treponemal RDT before a trep/non-trep RDT) in terms of cost and cost-effectiveness, relative to a single-assay combined testing strategy (using the trep/non-trep RDT alone), for two use cases: individual diagnosis and community surveillance.

**Methods:**

We use cohort decision analysis to examine the diagnostic and cost outcomes. We estimate cost and cost-effectiveness of the alternative testing strategies at different levels of prevalence of past/current infection and current infection under each use case. We take the perspective of the global yaws eradication programme. We calculate the total number of correct diagnoses for each strategy over a range of plausible prevalences. We employ probabilistic sensitivity analysis (PSA) to account for uncertainty and report 95% intervals.

**Results:**

At current prices of the treponemal and trep/non-trep RDTs, the sequential strategy is cost-saving for individual diagnosis at prevalence of past/current infection less than 85% (81–90); it is cost-saving for surveillance at less than 100%. The threshold price of the trep/non-trep RDT (below which the sequential strategy would no longer be cost-saving) is US$ 1.08 (1.02–1.14) for individual diagnosis at high prevalence of past/current infection (51%) and US$ 0.54 (0.52–0.56) for community surveillance at low prevalence (15%).

**Discussion:**

We find that the sequential strategy is cost-saving for both diagnosis and surveillance in most relevant settings. In the absence of evidence assessing relative performance (sensitivity and specificity), cost-effectiveness is uncertain. However, the conditions under which the combined test only strategy might be more cost-effective than the sequential strategy are limited. A cheaper trep/non-trep RDT is needed, costing no more than US$ 0.50–1.00, depending on the use case. Our results will help enhance the cost-effectiveness of yaws programmes in the 13 countries known to be currently endemic. It will also inform efforts in the much larger group of 71 countries with a history of yaws, many of which will have to undertake surveillance to confirm the interruption of transmission.

## Author summary

Yaws is a non-venereal treponemal infection. The disease is targeted by WHO for eradication by 2020. Testing is envisaged for diagnosis to confirm of clinical cases during treatment campaigns and for surveillance to certify the interruption of transmission. However resources available to the global eradication programme are severely limited and the cost of testing must be contained. Testing requires simultaneous detection of antibodies to both treponemal and non-treponemal antigens for diagnosis of active infection. Currently, there is one commercially available rapid diagnostic test for yaws that can do just that. However, it is considerably more expensive than the available syphilis tests detecting treponemal antibodies only. We evaluate the cost and cost-effectiveness of a sequential testing strategy (using the treponemal test first, before the combined test), relative to a combined testing strategy (using only the combined test). We consider the two use cases: individual diagnosis and community surveillance. We find that the sequential strategy is cost-saving for both diagnosis and surveillance in most relevant settings. Yaws eradication programme should consider adopting the sequential strategy. Still, a cheaper trep/non-trep RDT is needed, costing no more than US$ 0.50–1.00. Our results will help enhance the cost-effectiveness of yaws programmes in the 13 countries known to be currently endemic. It will also inform efforts in the much larger group of 71 countries with a history of yaws, many of which will have to undertake surveillance to confirm the interruption of transmission.

## Introduction

Yaws is a non-venereal treponemal infection caused by *Treponema pallidum* subspecies *pertenue* affecting primarily the skin in the early stages and the bone and cartilage in the late stages. In 1950, WHO estimated that 160 million people were infected with yaws. Between 2008 and 2012 more than 300 000 new cases were reported to the World Health Organization (WHO). The disease is now targeted by WHO for eradication by 2020. One or two rounds of mass treatment at high levels of population coverage have been shown to reduce prevalence of yaws near to elimination levels.[1] This approach is known as total community treatment (TCT)–treatment of an entire endemic community irrespective of the number of active clinical cases. A second important element of the WHO strategy is 6 monthly Total Targeted Treatment (TTT)–treatment of all active clinical cases and their contacts—to mop-up cases missed in TCT rounds.

Confirmation of clinical cases during TTT programs may be carried out using a rapid diagnostic test (RDT) for the dual detection of treponemal and non-treponemal serological markers at or near to point-of-care. Serological testing is also envisaged for certification of the interruption of transmission of *T*. *p pertenue*.

Yaws and syphilis treponemes differ in less than 0.2% of the genome sequence.[2] Yaws is serologically indistinguishable from syphilis, caused by *T pallidum* subspecies *pallidum*.[3] Serological tests developed for syphilis may therefore be used to diagnose yaws, especially among children, since its clinical manifestation and epidemiology differ from that of syphilis and may allow a differentiation of the two conditions. Serological diagnosis of clinically active yaws requires the detection of two distinct sets of antibodies: one against treponemal antigens and one against non-treponemal antigens. Treponemal in vitro diagnostics (IVDs), including *T*. *pallidum* particle agglutination assay (TPPA), *T*. *pallidum* hemagglutination assay (TPHA), and fluorescent treponemal antibody absorption test (FTA-ABS) are highly sensitive and specific but antibodies remain detectable for life following any treponemal infection even after successful treatment. A reactive treponemal test result can therefore indicate either current or past infection and may not be sufficient to indicate no new disease in people with clinical symptoms that look like yaws.

Non-treponemal IVDs, including Rapid Plasma Reagin (RPR) and Venereal Disease Research Laboratory (VDRL) assays, are less specific but since titers rise during active disease and fall following treatment, current and past infection can be distinguished. Titers refer to how many serial dilutions you can perform on the sample and still get a positive result. False positive results can occur when using non-treponemal assays alone due to acute viral infections, malaria, and connective tissue diseases which may also cause non-treponemal assays to be reactive. As a result, testing for yaws requires both treponemal and non-treponemal assays to give an accurate diagnosis of current yaws infection.

The most widely recommended yaws screening tool is the laboratory-based RPR followed by a treponemal test. RPR requires laboratory capacity, trained laboratory personnel, refrigeration for storage of reagents, and electricity to run equipment such as the refrigerator, centrifuge, and shaker. Because such facilities are generally not available in the remote areas where yaws is commonly endemic, diagnosis is often made on the basis of clinical findings only which may not be adequate for surveillance purposes. In places where laboratories are able to do the RPR, serum specimens have to be transported to centralized laboratories for testing and results are available in days or weeks. This delay may result in delayed treatment and continued transmission of the disease.

Rapid syphilis tests detecting treponemal antibodies (treponemal RDTs) are now commercially available, meeting minimum defined standards for quality, safety and performance for use at point-of-care. Treponemal RDTs have been introduced into national antenatal care programmes but these are not commonly used for yaws, as results of treponemal RDTs alone correlate poorly with presence of current infection, as explained earlier.

Currently, one commercially available RDT exists that is based on the simultaneous detection of antibodies to both treponemal and non-treponemal antigens. The DPP Yaws Trep & N.Trep Assay (Chembio, Medford, NY, USA) is designed for use in resource-limited settings where there is limited access to laboratory facilities. For brevity, we refer generically to the assay as a treponemal/non-treponemal RDT or “trep/non-trep RDT”. The dual components of the assay allows clinicians to both screen and confirm the serological status within 15 minutes and allows for differentiation of current and past yaws.

In 2014, the use of trep/non-trep RDT for diagnosis of yaws infection was evaluated and compared with *T*. *pallidum* particle hemagglutination assay (TPHA) and RPR as reference standards for treponemal and non-treponemal antibodies detection, respectively.[4] In the low-resource setting of Papua New Guinea, the treponemal test line demonstrated a sensitivity of 88.4% and a specificity of 95.2%; the non-treponemal test line demonstrated a sensitivity of 87.9% and a specificity of 92.5%. A number of evaluations of a trep/non-trep RDT for the diagnosis of yaws infection have now been conducted, as synthesized in a recent meta-analysis.[5]

It is expected that the simpler trep/non-trep RDT should improve access to yaws diagnosis relative to the RPR test. However, use of the trep/non-trep RDT alone may not be the most economical option, especially in low treponemal test positive prevalence settings. In yaws elimination pilot projects, WHO had negotiated a price of US$ 2.50 per trep/non-trep RDT and US$0.45 per treponemal RDT. For surveys where large number of people are non-reactive to the treponemal test, such as in low endemicity settings, a combination of two rapid tests (treponemal RDT for screening, and trep/non-trep RDT for diagnosis) could be cost-saving.

Studies have reported that antenatal syphilis screening and treatment is highly cost-effective in low and middle income countries.[6] Some have modelled the cost-effectiveness of different screening strategies.[7][8][9][10] Terris-Prestholt et al. (2015) were the first to compare the full range of possible screening and treatment strategies for syphilis in multiple countries, including Peru, Tanzania and Zambia. This range included a sequential strategy using a treponemal RDT followed by a dual trep/non-trep RDT. They found that the dual-only strategy was significantly higher cost than the sequential strategy in all three countries, but resulted in more true cases being detected and treated, with the result that cost-effectiveness was about the same in two out of three countries, namely Tanzania and Zambia, where prevalence was highest.

No such economic evaluation of testing strategies has been done for yaws.

**Fig 1:**
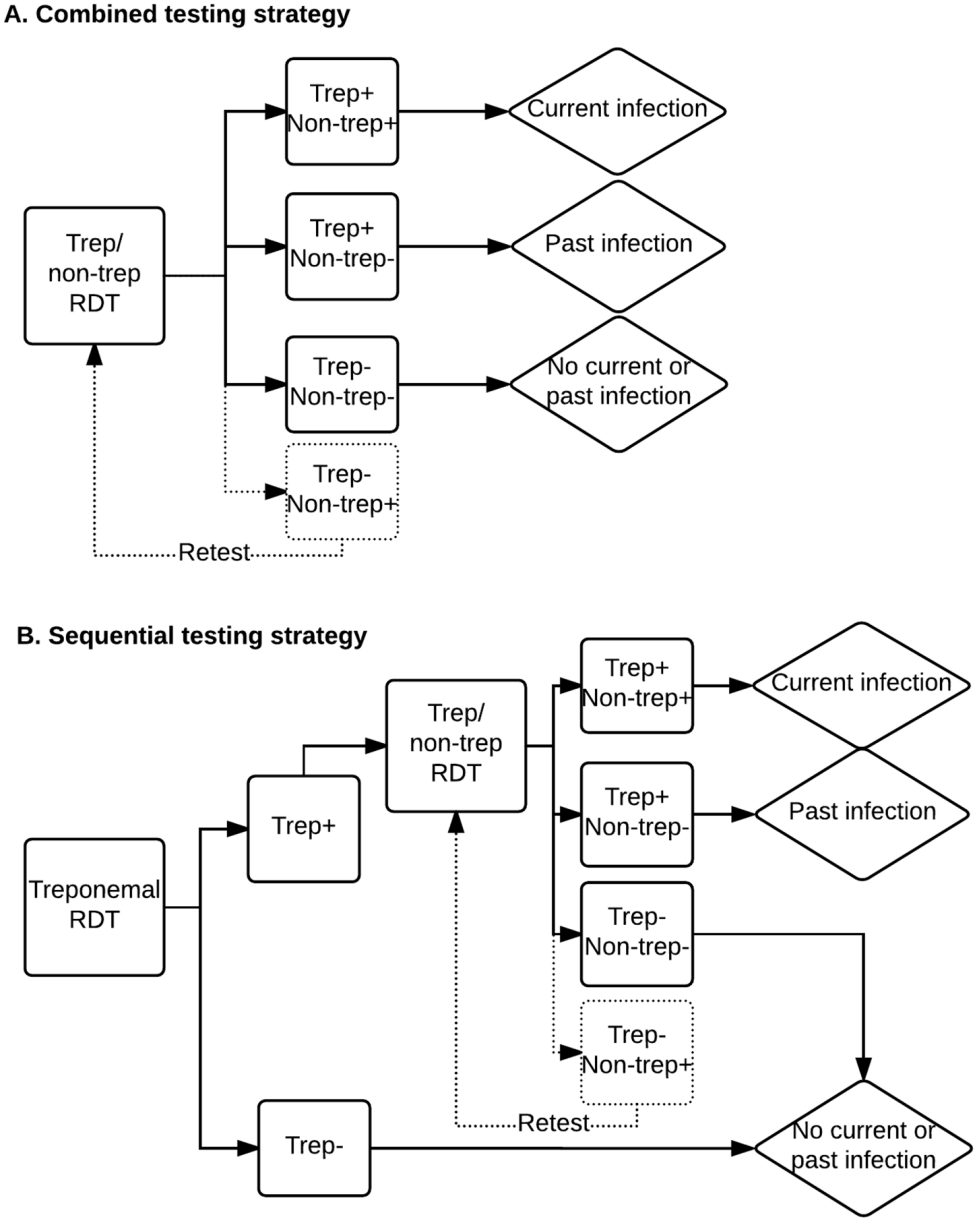
Diagram of alternative testing strategies: Combined (panel A) and sequential (panel B). Boxes represent tests and test results; diamonds represent diagnoses; dotted lines represent discordant treponemal and non-treponemal test results by the trep/non-trep RDT, excluded from Fig 2 for simplicity; in the case of discordant results between the treponemal RDT and the trep/non-trep RDT, the sequential testing strategy takes the result of the trep/non-trep RDT.

We therefore evaluate a two-assay sequential testing strategy in terms of both its cost and cost-effectiveness relative to a single-assay testing strategy. In the sequential strategy, a treponemal RDT is used as the screening assay of the testing strategy, followed by reflex testing with a trep/non-trep RDT for only the reactive treponemal specimens, as depicted in Fig 1. This strategy avoids unnecessary dual treponemal/non-treponemal testing of individuals with no past or current yaws infection (i.e. treponemal negative). The sequential testing strategy is compared to a single-assay testing strategy using the trep/non-trep RDT on the entire testing population.

We aim to establish the conditions (namely, prevalence of past/current infection and relative prices of the treponemal and trep/non-trep tests) under which the sequential strategy would be cost-saving or cost-effective relative to the combined strategy, for the purposes of 1) individual diagnosis and 2) community surveillance. By diagnosis, we mean confirmation of clinically suspected cases in individuals before TCT or during TTT; by surveillance we mean screening of communities (mostly asymptomatic individuals) for the purpose of verification of the interruption of transmission in population after TCT and in countries of historic endemicity.

## Methods

We use cohort decision analysis to examine the diagnostic and cost outcomes. We estimate cost and cost-effectiveness of the alternative testing strategies in a hypothetical testing population of 1000 people at different levels of past or current prevalence. We place these results in the context of treponemal positive and dually positive prevalences in Ghana, Papua New Guinea, Solomon Islands and Vanuatu—four endemic countries in which population serosurveys were undertaken in the years 2013–2014. These surveys were administered both pre- and post-TCT.

In estimating costs, we take the perspective of the global yaws eradication programme and national health systems. We include the cost of commodities to be funded in large part by the global yaws eradication programme, and the cost of other inputs such as labor to be supplied by the national health system.

We apply a unit cost of US$ 2.50 for each trep/non-trep RDT. For the sequential strategy, we apply a unit cost of US$ 0.45 for each treponemal RDT, and US$ 2.50 for each trep/non-trep RDT. We also add the cost of alcohol swabs ($3 for 100), sterile lancets ($375 for 2000) and non-sterile gloves ($3 for 50 pairs). These prices are consistent with the UNICEF supply catalogue.[11] These ancillary costs increase the unit cost of each trep/non-trep RDT and treponemal RDT to US$ 2.78 and US$ 0.73 respectively.

We consider that every test requires 2–5 minutes of a district-level laboratory technician’s time (depending on experience, this is the time it takes to collect the sample, execute the test, read and report the result). It takes 10–15 minutes between execution of the test and reading of its results, but technicians can attend to other patients during that time. We asked national yaws eradication programmes to provide estimates of the wage of a district-level laboratory technician (in US$). It ranged from US$ 210–510 per month in 11 of the 13 endemic countries, and US$ 1500–1585 per month in two small island developing states (Solomon Islands and Vanuatu).

In the sequential strategy, the trep/non-trep RDT is applied only to treponemal test positives (true and false positives). Total costs (and savings) therefore depend not only on the unit costs described above, but on the sensitivity and specificity of the treponemal RDT for yaws testing. All else equal, a less sensitive (specific) treponemal RDT will result in a smaller (larger) number of trep /non-trep RDTs required in the sequential testing strategy. We use sensitivity and specificity of the treponemal RDT from the Jafari et al. (2013) metanalysis.[12] Sensitivities and specificities are reported in Table 1. In probabilistic sensitivity analysis (PSA), we use the 95% confidence intervals for the sensitivity and specificity results.

**Table 1:** Reported sensitivities and sensitivities for DPP Yaws Trep & N.Trep assay and SD BIOLINE syphilis 3.0.

Test	Source	Disease	Sample subgroup	Line	Reference standard	Sensitivity, %(95% CI)	Specificity, % (95% CI)
SD BIOLINE Syphilis 3.0 (Standard Diagnostics, Inc.)	Calculated using data from Jafari et al 2013	Syphilis	Sexually transmitted infection clinics	Trep	TPHA	88.7 (85.1–91.8)	99.2 (98.7–99.5)
			Ante natal care (ANC)	Trep	TPHA	82.2 (79.7–84.6)	97.9 (97.5–98.2)
	Hypothetical*	Yaws	Primary and secondary disease	Trep	TPHA	85.3 (80.9–89.3)	95.8 (93.8–97.2)
			Asymptomatic	Trep	TPHA	56.9 (49.5–65.6)	97.7 (95.3–99.1)
DPP Yaws Trep & N.Trep Assay (Chembio Diagnostic Systems, Inc.)	Calculated using data from Marks et al 2016, combining primary and secondary disease	Yaws	Primary and secondary disease	Trep	TPHA	88.3 (85.4–90.8)	95.8 (93.8–97.2)
				Non-trep	RPR	86.7 (83.3–89.6)	94.7 (92.8–96.3)
			Asymptomatic	Trep	TPHA/TPPA	60.2 (51.1–68.7)	97.7 (95.3–99.1)
				Non-trep	RPR	43.1 (34.2–52.3)	95.5 (92.6–97.5)
		Syphilis	Primary and secondary disease	Trep	TPHA/TPPA	95.1 (91.5–97.5)	85.7 (57.2–98.2)
				Non-trep	RPR	96.3 (92.9–98.4)	57.1 (34.0–78.2)
			Asymptomatic	Trep	TPHA/TPPA	91.2 (88.5–93.5)	95.6 (93.8–98.6)
				Non-trep	RPR	94.7 (91.9–96.7)	67.1 (62.3–71.6)

CI—Confidence Interval. RPR—Rapid Plasma Reagin. TPHA—*Treponema pallidum* haemagglutination assay. TPPA–*Treponema pallidum* particle agglutination assay.

* Sensitivity of the treponemal RDT for yaws is allowed to be lower than that of the trep/non-trep RDT and specificity is assumed to be equivalent.

Using the Jafari et al (2013) data, we calculate sensitivity and specificity of the treponemal RDT for two relevant subgroups: clinical syphilis cases to be confirmed at sexually transmitted infection clinics, and (asymptomatic) pregnant women to be screened at ante natal care (ANC) clinics. Unfortunately, the results from Jafari et al. (2013) relate to syphilis testing only—there is no evidence of the performance of the treponemal RDT for yaws testing. Marks et al. (2016) found that the sensitivities of both components of the trep/non-trep RDT were higher in patients with syphilis than in patients with yaws at low titers, but not at high titers.[5] It is possible, if not probable, that the sensitivity of the treponemal RDT may therefore be worse for yaws than for syphilis.

We therefore adjust (downward) the sensitivity of the treponemal RDT by the ratio of the sensitivity of the trep/non-trep RDT for yaws to the sensitivity of the trep/non-trep RDT for syphilis. This adjustment, while crude, allows for the possibility that the sensitivity of the trepenomal RDT could be inferior to that of the treponemal line of the trep/non-trep RDT. In PSA, we allow the sensitivity of the treponemal RDT to vary between this adjusted number and that of the treponemal line of the trep/non-trep RDT. We assume that the specificity of the treponemal RDT for yaws is the same as that of the treponemal line of the trep/non-trep RDT. The hypothetical performance of the treponemal RDT for yaws is reported in Table 1.

We multiply unit costs by the total number of each test required. From total costs, we calculate the cost savings associated with sequential testing strategy. We then calculate the so-called threshold unit cost of the trep/non-trep RDT at which the sequential strategy would no longer be cost-saving, assuming a fixed price for the treponemal RDT. That is, we calculate the unit cost of the trep/non-trep RDT such that:

$$C_{d}\times P<C_{t}\times P+C_{d}\times P\times \left\{T_{p}\times Se_{t}+\left(1-T_{p}\right)\times \left(1-Sp_{t}\right)\right\}$$

And where:

*C*_*d*_ is the unit cost of the dual trep/non-trep RDT, including the price of the assay as well as ancillary costs;

*P* is the population to be tested;

*C*_*t*_ is the unit cost of the treponemal RDT, including the price of the assay as well as ancillary costs;

*T*_*p*_ is the prevelance of past/current infection in the testing population;

*Se*_*t*_ is the sensitivity of the treponemal RDT; and

*Sp*_*t*_ is the specificity of the treponemal RDT.

Simplifying and re-arranging, the sequential strategy is no longer cost-saving when:

$$C_{d}<\frac{C_{t}}{1-\left\{T_{p}\times Se_{t}+\left(1-T_{p}\right)\times \left(1-Sp_{t}\right)\right\}}$$

Or:

$$\frac{\left(C_{d}-C_{t}\right)}{C_{d}}<T_{p}\times Se_{t}+\left(1-T_{p}\right)\times \left(1-Sp_{t}\right)$$

That is, when the percentage difference in unit cost of the treponemal RDT relative to the trep/non-trep RDT is less than the percentage of cases that will test positive using the treponemal RDT, which includes both true and false positives. This reactivity rate is determined by the treponemal positive prevelance (*T*_*p*_) and sensitivity (*Se*_*t*_) and specificity (*Sp*_*t*_) of the treponemal RDT. At current prices of the trep/non-trep and treponemal RDTs, the reactivity rate would have to be more than about 74%. Of course, a low reactivity rate of the treponemal RDT, while leading to cost-savings, may not be cost-effective if it results in fewer correct diagnoses.

**Fig 2:**
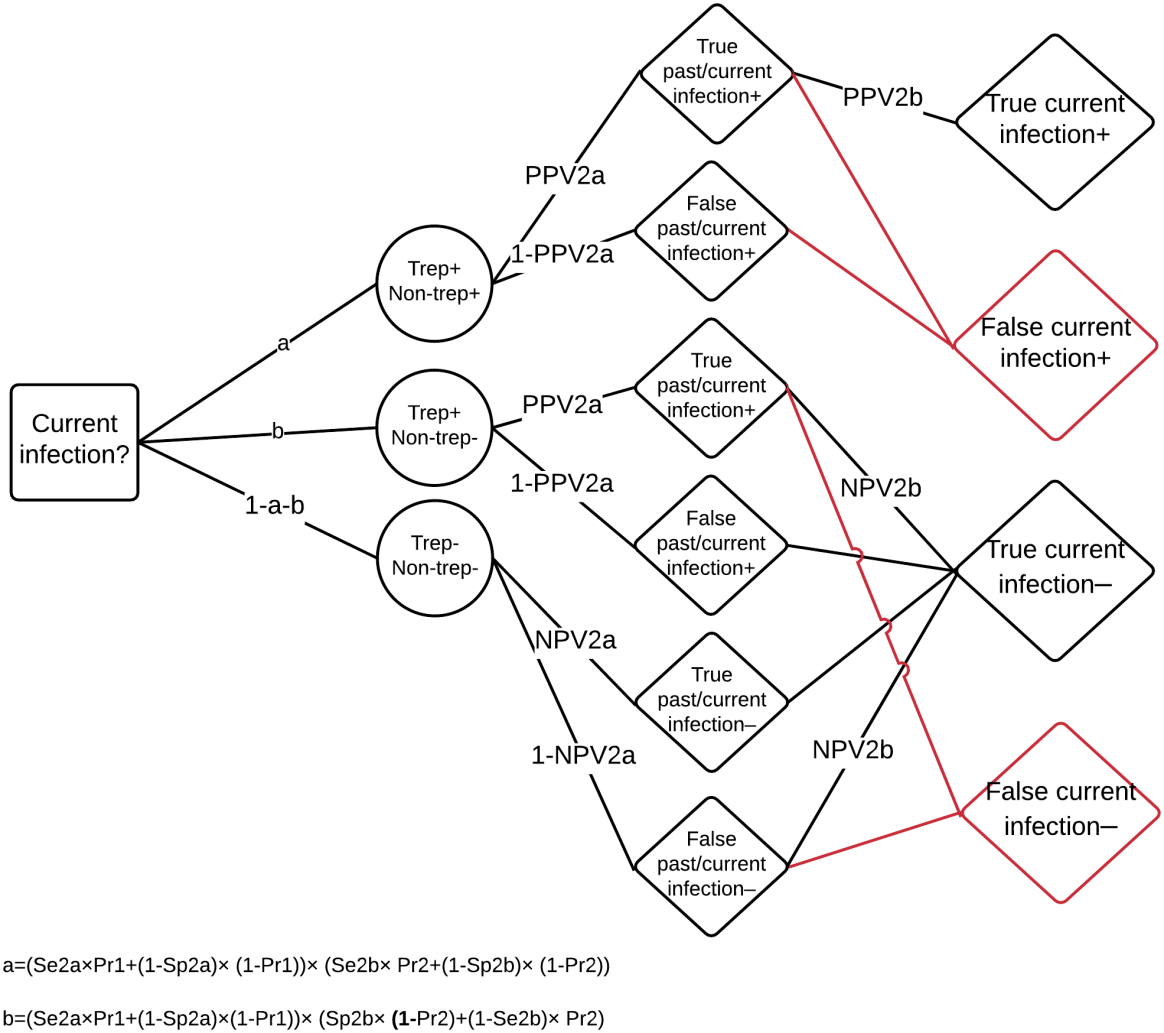
Decision model of diagnosis of current infection under the combined testing strategy. The decision to be made is whether an individual has current infection or not (box); the individual’s combined test result (circles) can be either: dually positive (suggesting current infection), treponemal positive only (past infection), or dually negative (never any infection); this depends on the Sensitivity (Se) and Specificity (Sp) of the treponemal line (Se2a and Sp2a, respectively) and of the non-treponemal line (Se2b and Sp2b), as well as on the prevalence of past/current infection in the total population (Pr1) and the prevalence of current infection in the population of past/current infections (Pr2); the treponemal line provides either a true or false diagnosis of past/current infection; this depends on Positive Predictive Value (PPV) and Negative Predictive Value (NPV) of the treponemal line (PPV2a and NPV2a, respectively); PPV is calculated as (Se×Pr1)÷(Se×Pr1+(1–Sp)×(1–Pr1)); NPV is calculated as Sp×(1–Pr1)÷((1-Se) ×Pr1+Sp×(1–Pr1)); the non-treponemal line provides either a true of false diagnosis of current infection; this depends on PPV and NPV of the non-treponemal line (PPV2b and NPV2b), using the prevalence of current infection in the population of past/current infections (Pr2); red lines indicate pathways to false diagnoses of current infection; note, the treponemal line can give a false positive diagnosis of past/current infection and yet the non-treponemal line can still give a correct overall diagnosis of no current infection.

**Fig 3:**
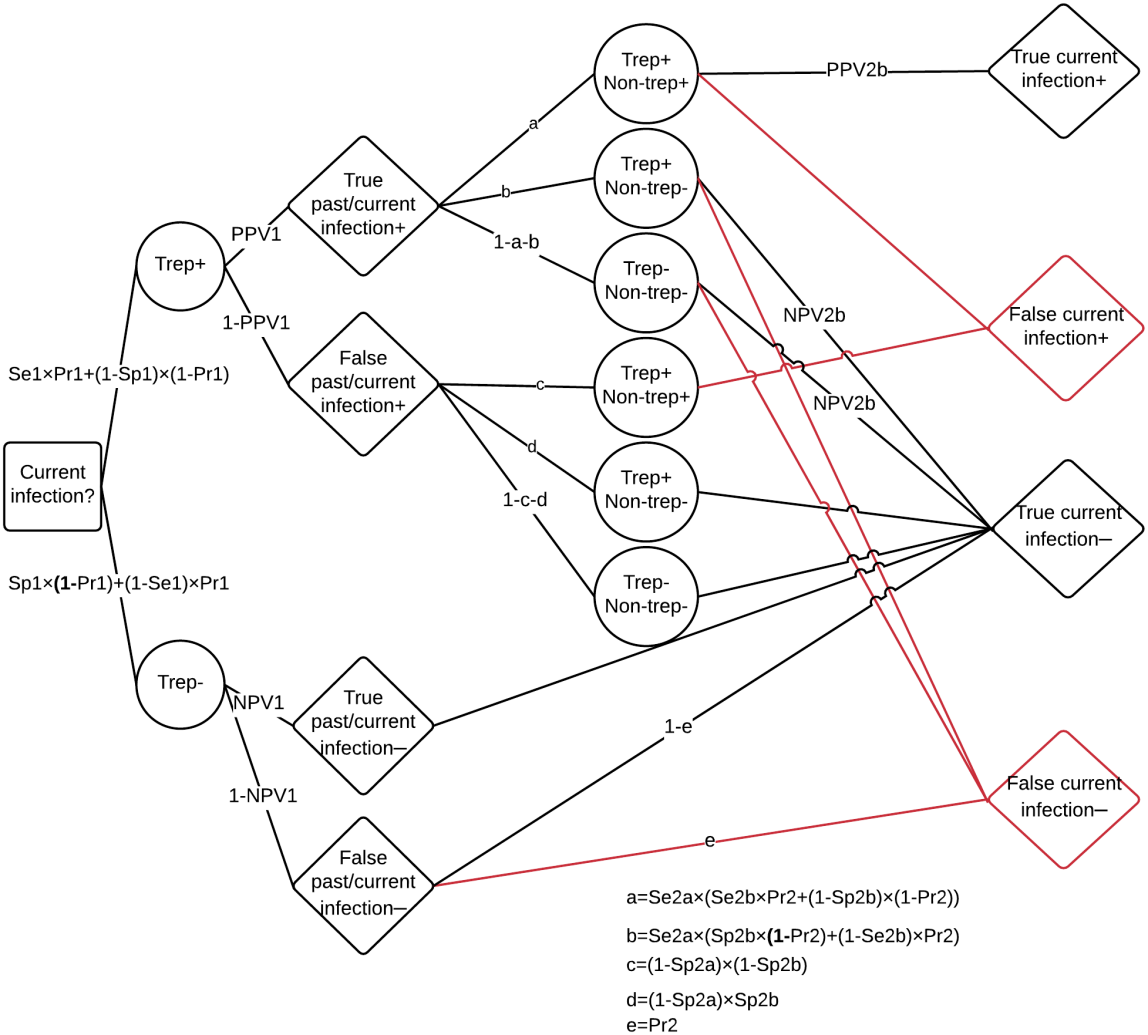
Decision model of diagnosis of current infection under the sequential testing strategy. In the sequential strategy, the individual’s first test result (circles) can be either: treponemal positive (suggesting past/current infection), or treponemal negative (never any infection); this depends on the Sensitivity (Se) and Specificity (Sp) of the treponemal RDT (Se1 and Sp1, respectively), as well as on the prevalence of past/current infection in the total population (Pr1); the treponemal line provides either a true or false diagnosis of past/current infection; this depends on Positive Predictive Value (PPV) and Negative Predictive Value (NPV) of the treponemal RDT (PPV1 and NPV1, respectively); PPV is calculated as (Se×Pr1)÷(Se×Pr1+(1–Sp)×(1–Pr1)); NPV is calculated as Sp×(1–Pr1)÷((1-Se) ×Pr1+Sp×(1–Pr1)); those with a true negative result for past/current infection are logically also truly negative for current infection; among those with a false negative result for past/current infection, we assume that the prevalence of current infection among false past/current infection negatives is the prevalence of current infection in the population of past/current infections (Pr2); those with either true or false positive results for past/current infection receive a second test; their second test result can be either: dually positive (suggesting current infection), treponemal positive only (past infection), or dually negative (never any infection); for simplicity, we assume that we use only the non-treponemal line; the non-treponemal line provides either a true of false diagnosis of current infection; this depends on PPV and NPV of the non-treponemal line (PPV2b and NPV2b),using the prevalence of current infection in the population of past/current infections (Pr2); red lines indicate pathways to false diagnoses of current infection.

We calculate the total number of correct diagnoses for each strategy over the full range of prevalences. Decision trees depicting the possible pathways to correct diagnosis are depicted for both strategies in Figs 2 and 3.

We assume that the percentage of past/current infections that are current is the same in the subset of true past/current infection positives identified by the treponemal RDT as it is in the total population of past/current infections (Fig 3). This assumption is thought to be reasonable; in Marks et al (2016), 74% of TPHA positive people had positive RPR; 75% of people with a positive treponemal RDT had a positive RPR, and 77% had a positive non-trep RDT. We also assume that the prevalence of current infection among false past/current infection negatives is (at most) equal to the prevalence of current infection among past/current infections; in any case, in an eradication programme, the number of false negatives will tend towards zero.

We use sensitivity and specificity of the trep/non-trep RDT from the Marks et al. (2016) meta-analysis. Performance characteristics depend on the use case of the trep/non-trep RDT: yaws diagnosis or yaws surveillance. In confirmation of clinical cases, more people will have high titres (where the test performs better) while in confirmation of the interruption of transmission more people will have low titres (where the test performs less well). We therefore consider performance characteristics for primary and secondary disease, or asymptomatic cases (Table 1). The former is applied to populations with clinical symptoms requiring diagnosis, while the latter is applied to populations requiring surveillance.

We calculate the cost per correct diagnosis (true current infection positive or negative) under each strategy (sequential or combined strategy) and use case (diagnosis or surveillance). However, we also report the cost per true positive diagnosis, considering that true positive and negative diagnoses may not be equivalent in their benefits. In the context of individual diagnosis for eradication, for example, true positive diagnosis may be more important than a true negative diagnosis, at least from the perspective of the health system. The incremental cost of treating a false positive is relatively trivial, even considering the cost attributed to any side effects. There are very few and minor side effects associated with azithromycin and indeed, many collateral benefits for diarrheal and other diseases. From the perspective of patients, however, there may be psychosocial costs associated with false positive results.

We then calculate the incremental cost-effectiveness ratio (ICER) of the higher cost combined strategy, for the range of treponemal and dually positive prevalences over which it is not dominated by the sequential strategy. By not dominated, we mean that while the cost is higher, the number of correct diagnoses is also higher. We present cost savings and cost-effectiveness of the alternative testing strategies in the context of survey population prevalences obtained in four countries: Ghana, Papua New Guinea (PNG), Solomon Islands, and Vanuatu.[13–15]

Pre-TCT survey population prevalences obtained using trep/non-trep RDTs are provided in Supporting Information S1 Table. Treponemal positive prevalence varied from 22% in Vanuatu to 51% in PNG. Among those testing treponemal positive, non-treponemal positives were between 21% in Solomon Islands and 71% in Vanuatu. Out of the total population tested, dually positive prevalences were between 7% in Solomon Islands and 18% in PNG.

Post-TCT survey population prevalences obtained using trep/non-trep RDTs are presented for four countries in Supporting Information S2 Table. The treponemal positive prevalence decreased to between 15% in Ghana and 42% in Solomon Islands. Among those testing treponemal positive, non-treponemal positives were between 5% in Solomon Islands and 49% in Vanuatu. The dually positive prevalence decreased, as a percentage of the population tested, to between 1% in Solomon Islands and 8% in Vanuatu.

We are not in this paper attributing these reductions in prevalence to TCT. We are simply using pre- and post-TCT prevalence as a proxy for the prevalence that one might encounter in community surveillance and individual diagnosis settings, respectively.

Use cases and prevalences of the testing population are not independent. In particular, prevalences will be higher when doing individual diagnosis than when doing community surveillance. We therefore focus on the following plausible ranges of prevalence: for individual diagnosis, current/past infection prevalence of 20–55%, of which 20–75% is currently infected; for community screening, current/past infection prevalence of 15–45%, of which 5–50% are currently infected.

We report best estimates using the median of 1000 simulations and the 95% confidence intervals using the 2.5^th^ and 97.5^th^ centiles. All data analysis and visualization were done using R (Foundation for Statistical Computing, Vienna, Austria).[16] All the necessary code is provided as Supporting Information.

## Results

We present results separately for the two use cases: individual diagnosis and community surveillance. The differences in results between use cases are driven by different values of sensitivity and specificity in populations with different clinical presentations (stages of disease).

We visualize results over the full range of possible prevalences of past and/or current infection of the testing populations but focus on the plausible ranges determined by treponemal and dually trep/non-trep positive prevalences in Ghana, Papua New Guinea (PNG), Solomon Islands, and Vanuatu.

### Individual diagnosis

At the current price of the treponemal RDT and a high prevalence of past/current infection of 51% (the treponemal positive prevalence in pre-TCT Papua New Guinea), we obtain a threshold unit cost for the trep/non-trep RDT of US$ 1.38 (1.31–1.46), including ancillary costs (i.e. swabs, lancets and gloves) and laboratory technician time, or US$ 1.08 (1.02–1.14) for the price of the assay alone. This is the unit cost below which the sequential strategy would no longer be cost-saving for individual diagnosis in a testing population where about one in two are or have been infected.

**Fig 4:**
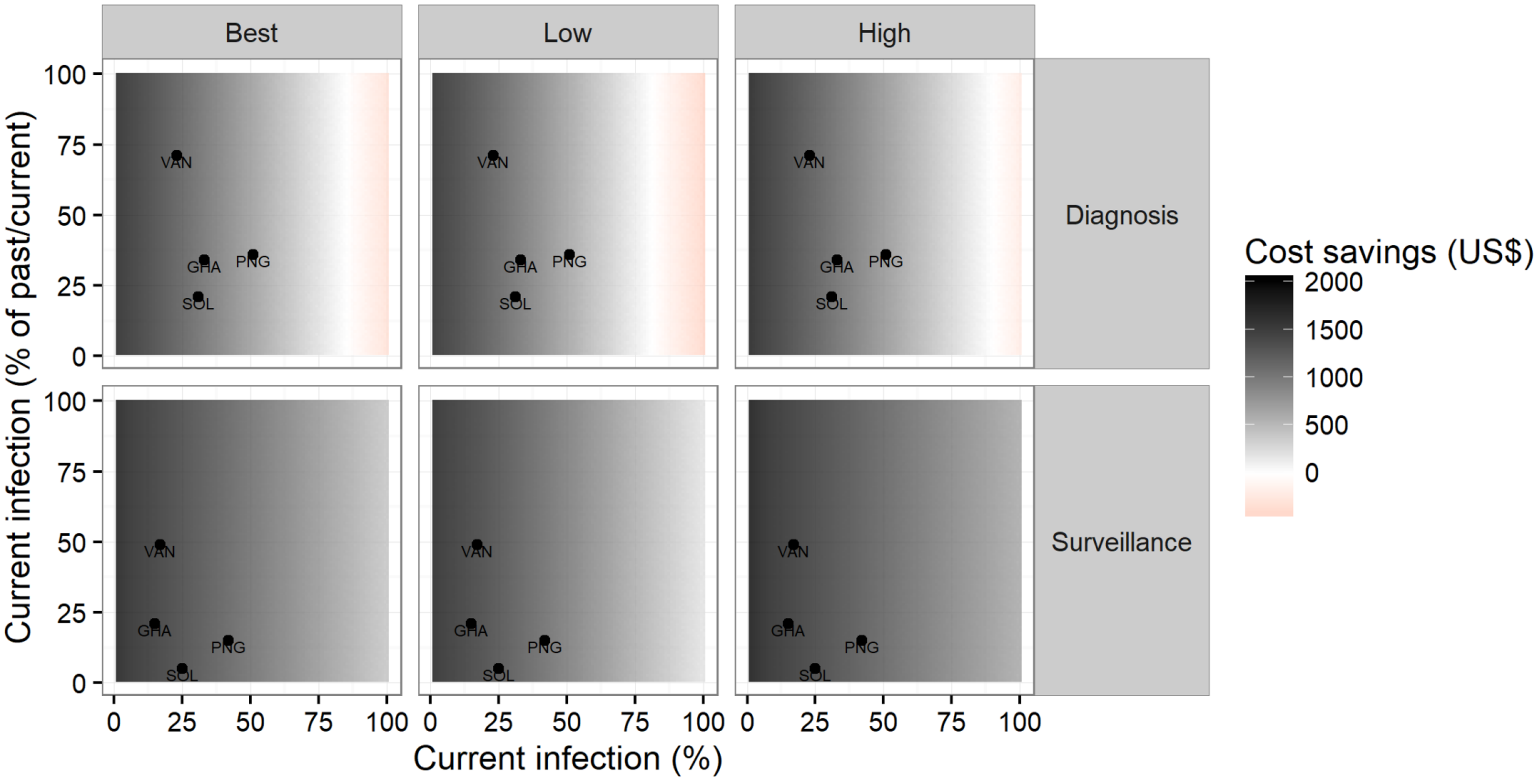
Cost savings of the sequential testing strategy across different scenarios of prevalence, per 1000 people tested, by use case. Best—best estimate (median); Low—low estimate (2.5th centile); High—high estimate (97.5th centile); cost savings are expressed per 1000 people tested.

More generally, costs savings of the sequential strategy in diagnosing 1000 individuals are presented in Fig 4 (top row) across all scenarios of prevalence. At current prices of the treponemal and trep/non-trep RDTs, the sequential strategy is cost-saving if the prevalence of past/current infection of the testing population is less than 85% (81–90). Within the plausible range of prevalence (20–55%), the savings are US$ 1079 (703–1448) per 1000 people tested. Above 85%, it is the combined strategy that is cost-saving.

The number of correct diagnoses of current infection (true positives and true negatives) is presented in Supporting Information S1 Fig (top two rows). Based on our assumptions about the relative performance of the treponemal RDT for yaws, the number of correct diagnoses is somewhat higher under the combined strategy than under the sequential strategy. However, in the plausible range of prevalences, more than 900 correct diagnoses are made for every 1000 people tested under both strategies. It is only at higher prevalences that differences between the strategies become non-trivial. The number of true current infection positives is presented in Supporting Information S2 Fig.

**Fig 5:**
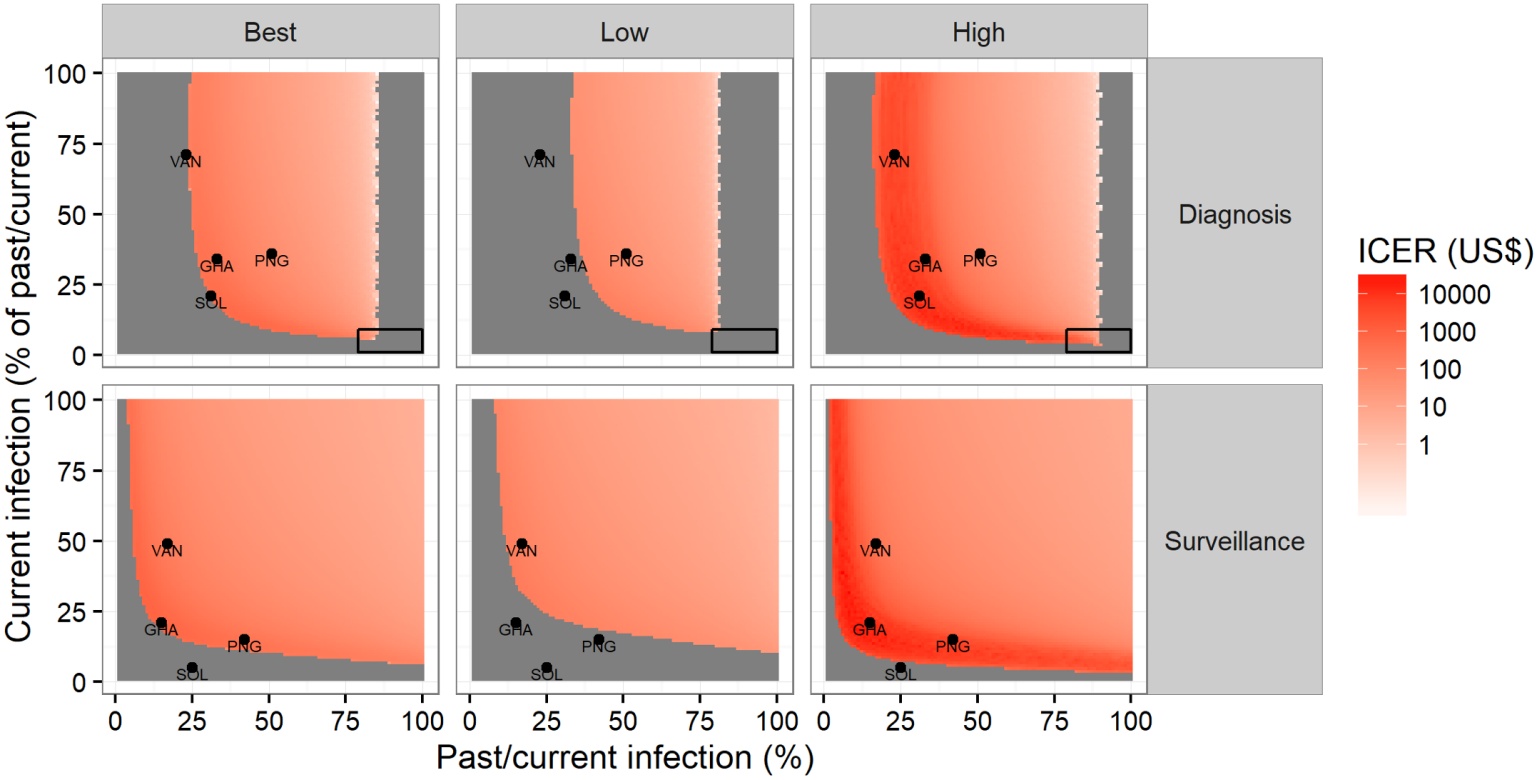
Incremental cost-effectiveness (cost per correct diagnosis gained) of the combined testing strategy across different scenarios of prevalence, by use case. Best—best estimate (median); Low—low estimate (2.5th centile); High—high estimate (97.5th centile); ICER—Incremental Cost Effectiveness Ratio (cost per correct diagnosis); black rectangles indicate where the sequential strategy may be more costly and more effective; grey areas without an ICER value indicate negative ICERs, where the combined testing strategy is less effective and more costly or more effective and less costly.

Given our assumptions about the relative performance of the treponemal RDT, there is a range of prevalences over which a higher cost and (hypothetically) more sensitive combined strategy could be more cost-effective than the sequential strategy. Incremental cost-effectiveness ratios (ICERs; ratio of incremental costs over incremental benefits or incremental cost per correct diagnosis gained) are presented in Fig 5 (top row), across different scenarios of prevalence. At prevalence of past/current infection of 51% and current infection of 18% (again, the trep/non-trep RDT positive prevalences in pre-TCT Papua New Guinea), the ICER is US$ 58 (42–103) per correct diagnosis gained.

At very high prevalence of past/current infection, where it becomes cost-saving, a more sensitive combined strategy may dominate the sequential strategy. At very low prevalence of either past/current infection or current infection, it is specificity that matters more for the number of correct diagnoses, and even a more sensitive combined strategy may be dominated by the sequential strategy. In theory, there is a combination of prevalences (very high prevalence of past/current infection and very low prevalence of current infection) where a more sensitive combined strategy could produce fewer correct diagnoses of current infection (this is the area depicted by a black rectangle in Fig 5). In practice, however, this combination is unlikely.

In Supporting Information S3 Fig (top row), we present the same figure, but using only true positive diagnoses in the denominator of the ICER. Here, the ICER is US$ 38 (32–48) at prevalence of past/current infection of 51% and current infection of 18%. Given our assumptions about the relative performance of the treponemal RDT, the combined strategy is nowhere dominated by the sequential strategy when considering only true positive diagnoses; the combined strategy dominates the sequential strategy wherever it is cost-saving.

The cost-effectiveness plane is presented in Supporting Information S4 Fig (top row), at the lower and upper limits of the plausible range of prevalence: for individual diagnosis, the current/past infection prevalence ranges from 20% (lower limit) to 55% (upper limit), of which 20% (lower limit) or 75% (upper limit) are currently infected. It shows that at the lower limit, the combined testing strategy is less effective in spite of being more costly; at the upper limit it results in somewhere between 20–60 additional correct diagnoses (per 1000 tested) for somewhere between US$ 500–600.

### Community surveillance

At the current cost of the treponemal RDT and a low prevalence of past/current infection of 15% of the testing population (similar to Ghana post-TCT), we obtain a threshold unit cost for the trep/non-trep RDT of US$ 0.84 (0.81–0.88), including ancillary costs and laboratory technician time, or US$ 0.54 (0.52–0.56) for the price of the assay alone.

At current prices of the treponemal and trep/non-trep RDTs, the sequential strategy is cost-saving in surveillance at all levels of prevalence of past/current infection—see Fig 4 (bottom row). Within the plausible range of prevalence (15–45%), the savings are US$ 1527 (1279–1748) per 1000 population.

The number of correct diagnoses of current infection (true positives and true negatives) is presented in Supporting Information S1 Fig (bottom two rows). Again, based on our assumptions about the relative performance of the treponemal RDT for yaws, the number of correct diagnoses is somewhat higher under the combined strategy than under the sequential strategy. Again, under both strategies, in the plausible range of prevalences, more than 900 correct diagnoses are made for every 1000 people tested.

ICERs are presented in Fig 5 (bottom row). At a prevalence of past/current infection of 42% and prevalence of current infection of 6% (similar to post-TCT Papua New Guinea), the best estimate is US$ 355 per correct diagnosis gained by the combined strategy. However, the low estimate is in an area of the plot where the combined strategy is dominated by the sequential strategy. Again, at very low prevalence of either past/current infection or current infection, it is specificity that matters more for the number of correct diagnoses.

In Supporting Information S3 Fig (bottom row), we present the same figure, but using only true positive diagnoses in the denominator of the ICER. Here, the ICER is US$ 117 (90–155) at a prevalence of past/current infection of 42% and prevalence of current infection of 6%. Given our assumptions about the relative performance of the treponemal RDT, the combined strategy is nowhere dominated by the sequential strategy when considering only true positive diagnoses; but, unlike in the diagnosis use case, the combined strategy is never cost-saving and nowhere dominates the sequential strategy.

The cost-effectiveness plane is presented in Supporting Information S4 Fig (bottom row), again at the lower and upper limits of the plausible range of prevalences: for community surveillance, current/past infection prevalence ranges from 15% (lower) to 45% (upper), of which 5% (lower) to 50% (upper) are currently infected.

## Discussion

In summary, this study finds that, at current prices, a sequential strategy is cost-saving relative to use of a combined strategy for individual diagnosis, at a prevalence of past/current infection less than 85% (81–90); it is cost-saving for community surveillance at a prevalence of less than 100% (i.e. always). The threshold prevalence for community surveillance is so high because when titres are low, the reactivity rate of the treponemal RDT is so low and so few people will need a non-treponemal result.

It turns out that the sequential strategy is no longer cost-saving for individual diagnosis in testing populations with high prevalence of past/current infection (i.e. 51%) when the price of the trep/non-trep RDT is less than US$ 1.08 (1.02–1.14). Likewise, the sequential strategy is no longer cost-saving for community surveillance in populations with low prevalence of past/current infection (i.e. 15%) when the price of the trep/non-trep RDT is less than US$ 0.54 (0.52–0.56).

In the absence of evidence assessing relative performance (sensitivity and specificity), the cost-effectiveness of a hypothetically more sensitive combined strategy is uncertain. However, the conditions under which it might be cost-effective are fairly limited. This finding is true even under fairly pessimistic assumptions about the performance of the treponemal RDT for yaws.

In addition to its relatively high cost, a major limitation of the current trep/non-trep RDT is its reduced sensitivity for low titer yaws, at least in the Solomon Islands where it was tested. Further research is required to determine whether available treponemal RDTs (for syphilis) perform any better for low titer yaws. Reduced sensitivity is likely to be a greater problem when using the test as part of yaws surveillance; a higher sensitivity assay will be needed to confirm interruption of transmission, such as RPR or even polymerase chain reaction (PCR) as PCR positive and trep/non-trep RDT negative cases have been observed. Criteria for eradication of yaws in the Morges strategy of 2012 are: 1) absence of new indigenous cases for 3 consecutive years; 2) absence of evidence of transmission for 3 continuous years measured with sero-surveys among children aged 1–5 years (for example, no young children with RPR sero-reactivity); and 3) negative PCR for *Treponema pallidum subspecies pertenue* in suspected lesions.[17]

There are several limitations to this study.

Serology does not result in identification of all cases of current yaws where early infection may be seronegative, and seropositive patients could have persisting antibodies after successful treatment. Therefore PCR is now considered the gold standard for the diagnosis of active yaws. The sensitivity and specificity of both the treponemal RDT and trep/non-trep RDT have not been assessed relative to PCR. However, there is no reason to believe that the bias favours the sequential testing strategy, as both the treponemal and trep/non-trep RDTs have been assessed against the same standard.

As described in the methods, treponemal RDTs have not been assessed for yaws, and we have therefore had to infer sensitivity and specificity from test performance for syphilis, as reported by Jafari et al (2013). Performance in syphilis is likely to be better than it is in yaws, as the trep/non-trep RDT also performs better in syphilis than in yaws. Although titres are often higher in syphilis compared with yaws (especially asymptomatic disease), it is unclear why Marks et al (2016) found that trep/non-trep performance was worse for yaws even when controlling for titre. Again, yaws and syphilis treponemes differ in less than 0.2% of the genome sequence.[2] Notwithstanding, that the specificity for yaws will be equal to or lower than that reported for syphilis should possibly be further assessed.

More generally, it should be noted that reported sensitivities and specificities can depend upon contextual factors, at least partially, and therefore the results of the meta-analyses of both Jafari et al (2013) and Marks et al (2016) may not fully reflect test performance in all settings, which underscores the need of interpreting our results in the light of the sensitivity analysis we performed.

Our probabilistic sensitivity analysis was focused on uncertainty around the relative performance of the tests, and to a lesser extent on costs. We assumed that the cost of traded commodities, procured by the global yaws eradication programme from international markets, was deterministic. Furthermore, we had only one estimate per country for the wage of laboratory technicians at the district level. In settings where either the commodity or labor costs are highly uncertain and/or their distribution highly skewed, a more sophisticated analysis of costs could be warranted.

We have not considered the time and other indirect costs incurred by the tested populations. The treponemal RDT produces results after 10 minutes; the trep/non-trep RDT requires 15 min. Under the sequential strategy, therefore, treponemal negatives wait 5 fewer minutes and treponemal positives wait 10 more minutes. Had we taken these costs into account, the results might have been less favourable to the sequential testing strategy in higher treponemal positive settings. Therefore, from a patient’s perspective too, there is a case to be made for negotiating lower prices for the trep/non-trep RDT in settings with a high prevalence of past/current infection.

A cheaper trep/non-trep RDT is needed, costing no more than US$0.50–1.00, depending on the use case.

However, other strategies are theoretically possible. RPR is already available and the centralized execution and availability of results may not be a major problem in some settings. Furthermore, a non-trep point of care RDT (alone, without the treponemal RDT) is technically feasible but has not yet been developed. An alternative strategy could involve the treponemal RDT followed by either RPR or the non-trep RDT. A reverse sequential strategy (non-trep test followed by the trep test) could also be possible.

Of course, these alternative sequential strategies not considered in our analysis would only be cost-saving relative to our original sequential strategy as long as the price of the RPR or non-trep RDT did not exceed the cost of the trep/non-trep RDT. Unfortunately, the cost of RPR, including transport to centralized or even international laboratories, will be prohibitively high in most of the settings in question, and we know of no plans to manufacture a non-trep point of care RDT.

Diagnosis and surveillance are essential to the yaws eradication effort. However, the yaws eradication effort is yet to be funded.[18] There are two situations of particular relevance in which savings could be substantial if the sequential testing strategy was implemented: first, during mass screening campaigns, before and after TCT; second, during final screening campaigns, including verification of the interruption of transmission. Cost savings from the sequential strategy could be reallocated to other essential interventions, such as sensitization to increase treatment coverage.

Our results will help enhance the cost-effectiveness of yaws programmes in the 13 countries known to be currently endemic. It will also inform efforts in the much larger group of 71 countries with a history of yaws, many of which will have to undertake surveillance to confirm the interruption of transmission.

## Supporting information

S1 Table Pre-TCT treponemal and dually positive survey population prevalence. GHA—Ghana; PNG—Papua New Guinea; SOL—Solomon Islands; VAN—Vanuatu; in Solomon Islands, post-TCT prevalence was assessed 6 months after TCT, whereas in other sites it was assessed 12 months after TCT; Ghana and Vanuatu used the trep/non-trep RDT, whereas Papua New Guinea and Vanuatu used the RPR with titre > 1:8; prevalences are therefore not directly comparable.
(DOCX)

S2 Table Post-TCT treponemal and dually positive survey population prevalence. GHA—Ghana; PNG—Papua New Guinea; SOL—Solomon Islands; VAN—Vanuatu; in Solomon Islands, post-TCT prevalence was assessed 6 months after TCT, whereas in other sites it was assessed 12 months after TCT; Ghana and Vanuatu used the trep/non-trep RDT, whereas Papua New Guinea and Vanuatu used the RPR with titre > 1:8; prevalences are therefore not directly comparable.
(DOCX)

S1 Fig Number of true diagnoses of current infection across different scenarios of prevalence, by use case and strategy. Best—best estimate (median); Low—low estimate (2.5th centile); High—high estimate (97.5th centile).
(TIFF)

S2 Fig Number of true current infection positives across different scenarios of prevalence, by use case and strategy. Best—best estimate (median); Low—low estimate (2.5th centile); High—high estimate (97.5th centile).
(TIFF)

S3 Fig Incremental cost-effectiveness (cost per true positive gained) of combined testing strategy across different scenarios of prevalence, by use case. Best—best estimate (median); Low—low estimate (2.5th centile); High—high estimate (97.5th centile); ICER—Incremental Cost Effectiveness Ratio (cost per correct diagnosis); grey areas without an ICER value indicate negative ICERs, where the combined testing strategy is less effective and more costly or more effective and less costly.
(TIFF)

S4 Fig Incremental costs and effects (correct diagnoses gained) of the combined testing strategy at the lower and upper limits of the plausible range of prevalence, by use case. Dots represent the 1000 simulated values from the probabilistic sensitivity analysis; for individual diagnosis, the current/past infection prevalence ranges from 20% (lower limit) to 55% (upper limit), of which 20% (lower limit) or 75% (upper limit) are currently infected; for community screening, current/past infection prevalence is 15–45%, of which 5–50% are currently infected; cost and effects are expressed per 1000 people tested.
(TIFF)
