# Supplementary material for: The cost and cost-effectiveness of rapid testing strategies for yaws diagnosis and surveillance
Source: PLoS Negl Trop Dis. 2017 Oct 26;11(10):e0005985. doi: 10.1371/journal.pntd.0005985 (PMC5658197; doi:10.1371/journal.pntd.0005985)
Supplement: S1 Table — GHA—Ghana; PNG—Papua New Guinea; SOL—Solomon Islands; VAN—Vanuatu; in Solomon Islands, post-TCT prevalence was assessed 6 months after TCT, whereas in other sites it was assessed 12 months after TCT; Ghana and Vanuatu used the trep/non-trep RDT, whereas Papua New Guinea and Vanuatu used the RPR with titre > 1:8; prevalences are therefore not directly comparable. (DOCX) [file pntd.0005985.s001.docx]

|  | **GHA** | | | **PNG** | | | **SOL** | | | **VAN** | | |
| --- | --- | --- | --- | --- | --- | --- | --- | --- | --- | --- | --- | --- |
|  | N | % | 95% CI | N | % | 95% CI | N | % | 95% CI | N | % | 95% CI |
| Total tested | 975 | 1.00 | – | 991 | 1.00 | – | 1679 | 1.00 | – | 997 | 1.00 | – |
| Trep positive | 322 | 0.33 | (0.3-0.36) | 508 | 0.51 | (0.48-0.54) | 519 | 0.31 | (0.29-0.33) | 226 | 0.23 | (0.2-0.25) |
| Trep negative | 653 | 0.67 | (0.64-0.7) | 483 | 0.49 | (0.46-0.52) | 1160 | 0.69 | (0.67-0.71) | 771 | 0.77 | (0.75-0.8) |
| Trep/non-trep dually positive | 108 | 0.11 | (0.09-0.13) | 181 | 0.18 | (0.16-0.21) | 110 | 0.07 | (0.05-0.08) | 161 | 0.16 | (0.14-0.18) |
