# Supplementary material for: The cost and cost-effectiveness of rapid testing strategies for yaws diagnosis and surveillance
Source: PLoS Negl Trop Dis. 2017 Oct 26;11(10):e0005985. doi: 10.1371/journal.pntd.0005985 (PMC5658197; doi:10.1371/journal.pntd.0005985)
Supplement: S2 Table — GHA—Ghana; PNG—Papua New Guinea; SOL—Solomon Islands; VAN—Vanuatu; in Solomon Islands, post-TCT prevalence was assessed 6 months after TCT, whereas in other sites it was assessed 12 months after TCT; Ghana and Vanuatu used the trep/non-trep RDT, whereas Papua New Guinea and Vanuatu used the RPR with titre > 1:8; prevalences are therefore not directly comparable. (DOCX) [file pntd.0005985.s002.docx]

|  | **GHA** | | | **PNG** | | | **SOL** | | | **VAN** | | |
| --- | --- | --- | --- | --- | --- | --- | --- | --- | --- | --- | --- | --- |
|  | N | % | 95% CI | N | % | 95% CI | N | % | 95% CI | N | % | 95% CI |
| Total tested | 1342 | 1.00 | – | 910 | 1.00 | – | 897 | 1.00 | – | 1100 | 1.00 | – |
| Trep positive | 199 | 0.15 | (0.13-0.17) | 386 | 0.42 | (0.39-0.46) | 228 | 0.25 | (0.23-0.28) | 184 | 0.17 | (0.14-0.19) |
| Trep negative | 1143 | 0.85 | (0.83-0.87) | 524 | 0.58 | (0.54-0.61) | 669 | 0.75 | (0.72-0.77) | 916 | 0.83 | (0.81-0.86) |
| Trep/non-trep dually positive | 41 | 0.03 | (0.02-0.04) | 59 | 0.06 | (0.05-0.08) | 11 | 0.01 | (0-0.02) | 90 | 0.08 | (0.07-0.1) |
