# Supplementary figures and images for: The cost and cost-effectiveness of rapid testing strategies for yaws diagnosis and surveillance
Source: PLoS Negl Trop Dis. 2017 Oct 26;11(10):e0005985. doi: 10.1371/journal.pntd.0005985 (PMC5658197; doi:10.1371/journal.pntd.0005985)

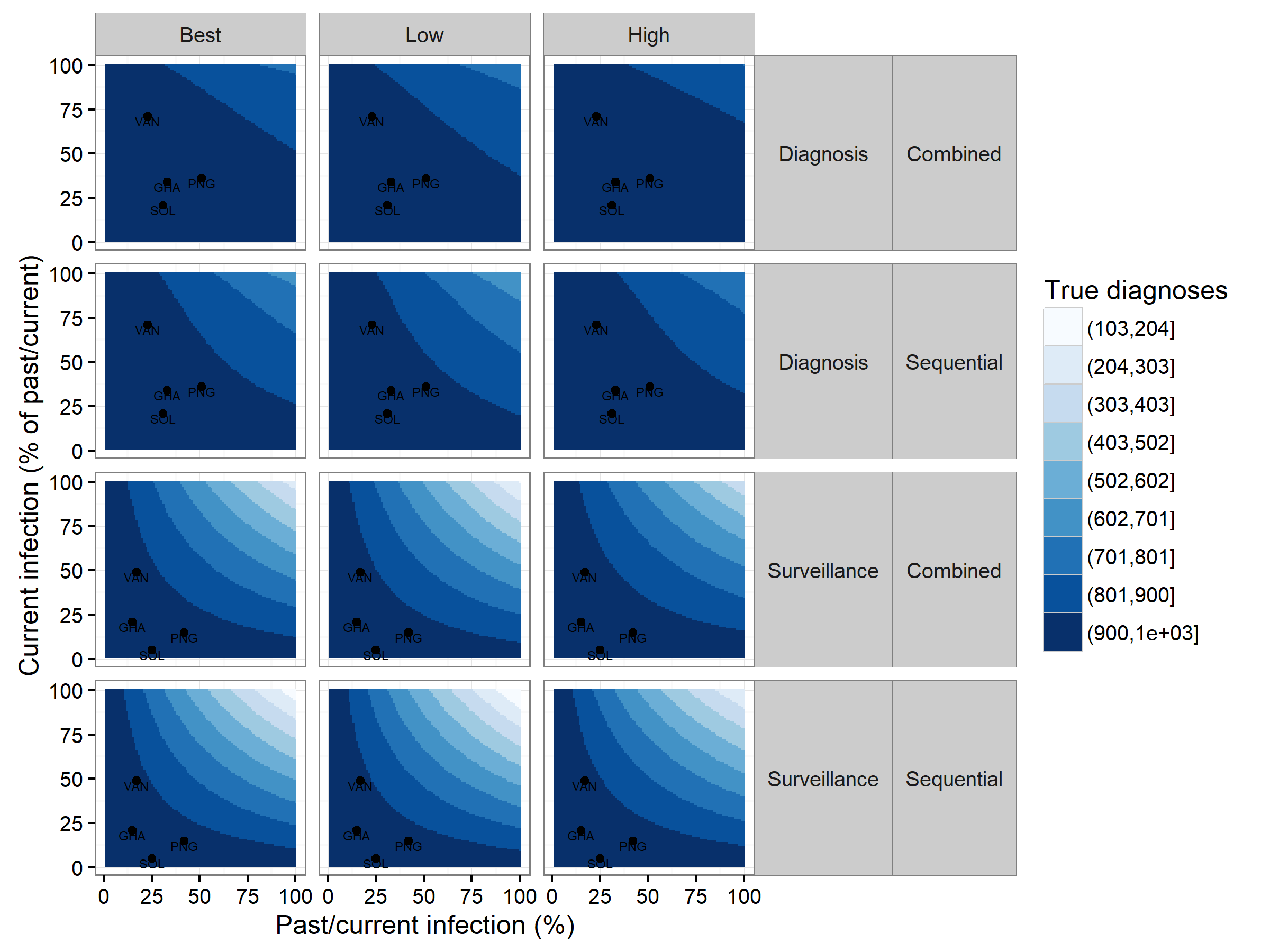

Supplement: S1 Fig — Best—best estimate (median); Low—low estimate (2.5th centile); High—high estimate (97.5th centile). (TIFF) [file pntd.0005985.s003.tiff]

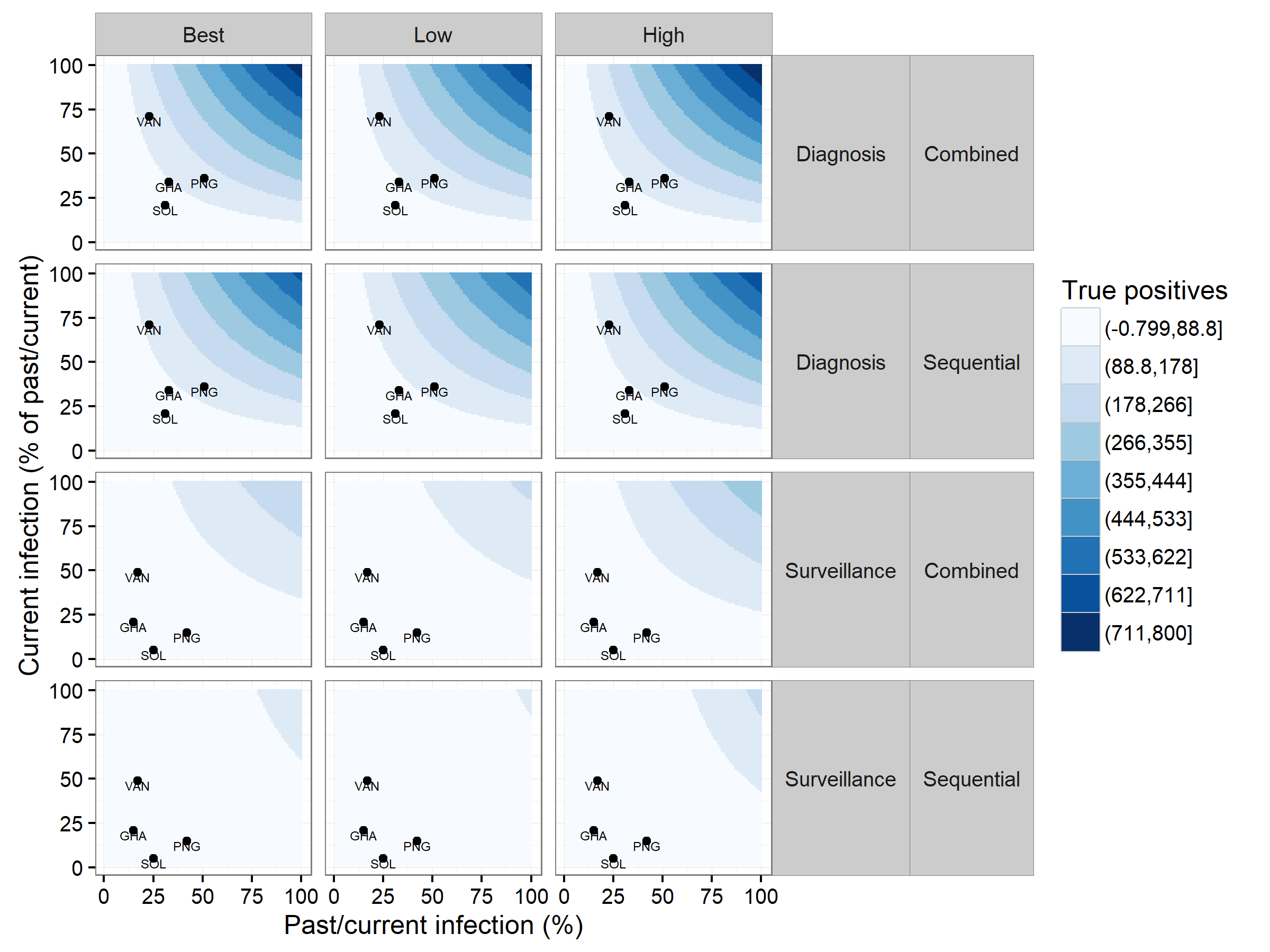

Supplement: S2 Fig — Best—best estimate (median); Low—low estimate (2.5th centile); High—high estimate (97.5th centile). (TIFF) [file pntd.0005985.s004.tiff]

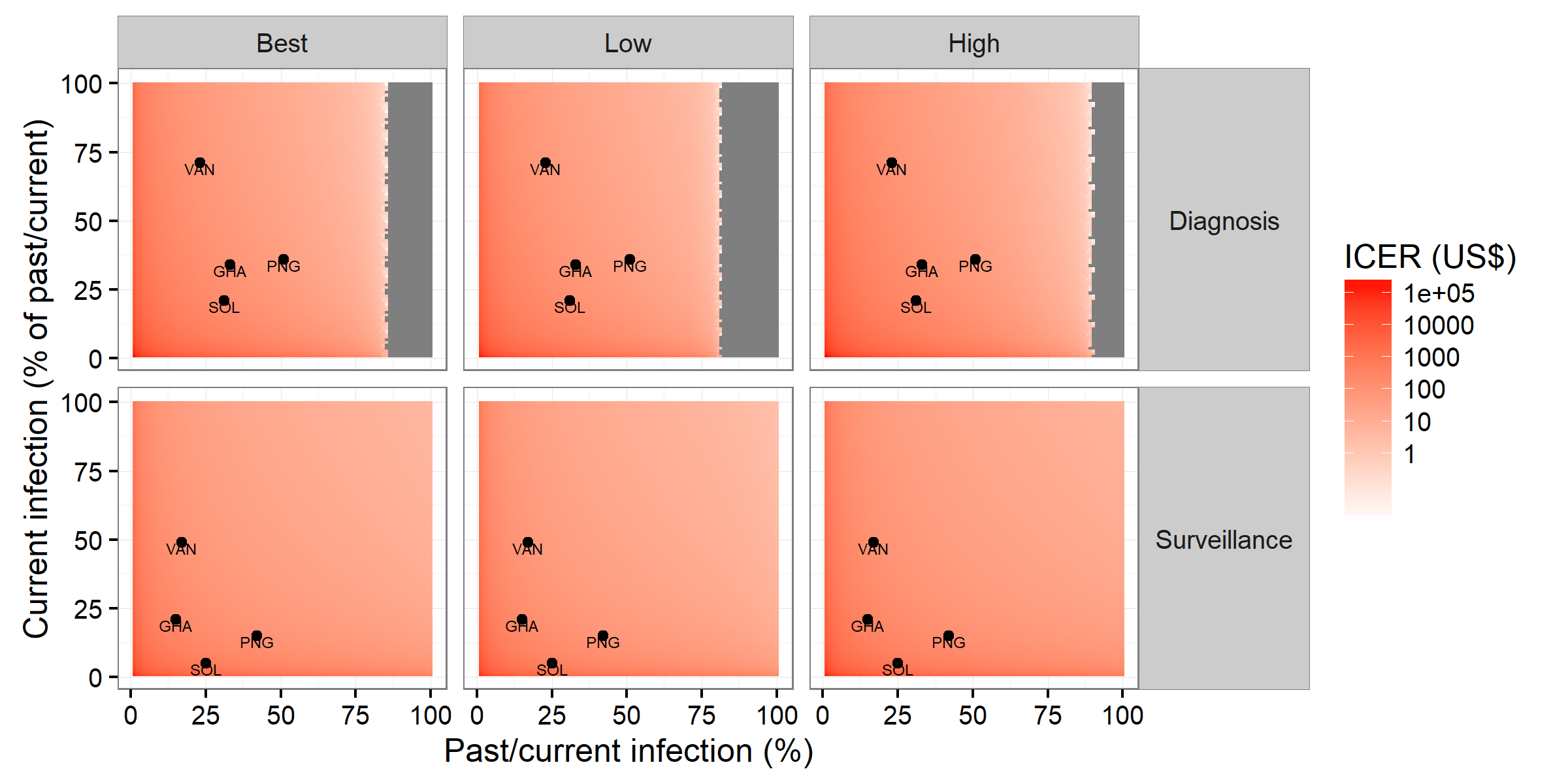

Supplement: S3 Fig — Best—best estimate (median); Low—low estimate (2.5th centile); High—high estimate (97.5th centile); ICER—Incremental Cost Effectiveness Ratio (cost per correct diagnosis); grey areas without an ICER value indicate negative ICERs, where the combined testing strategy is less effective and more costly or more effective and less costly. (TIFF) [file pntd.0005985.s005.tiff]

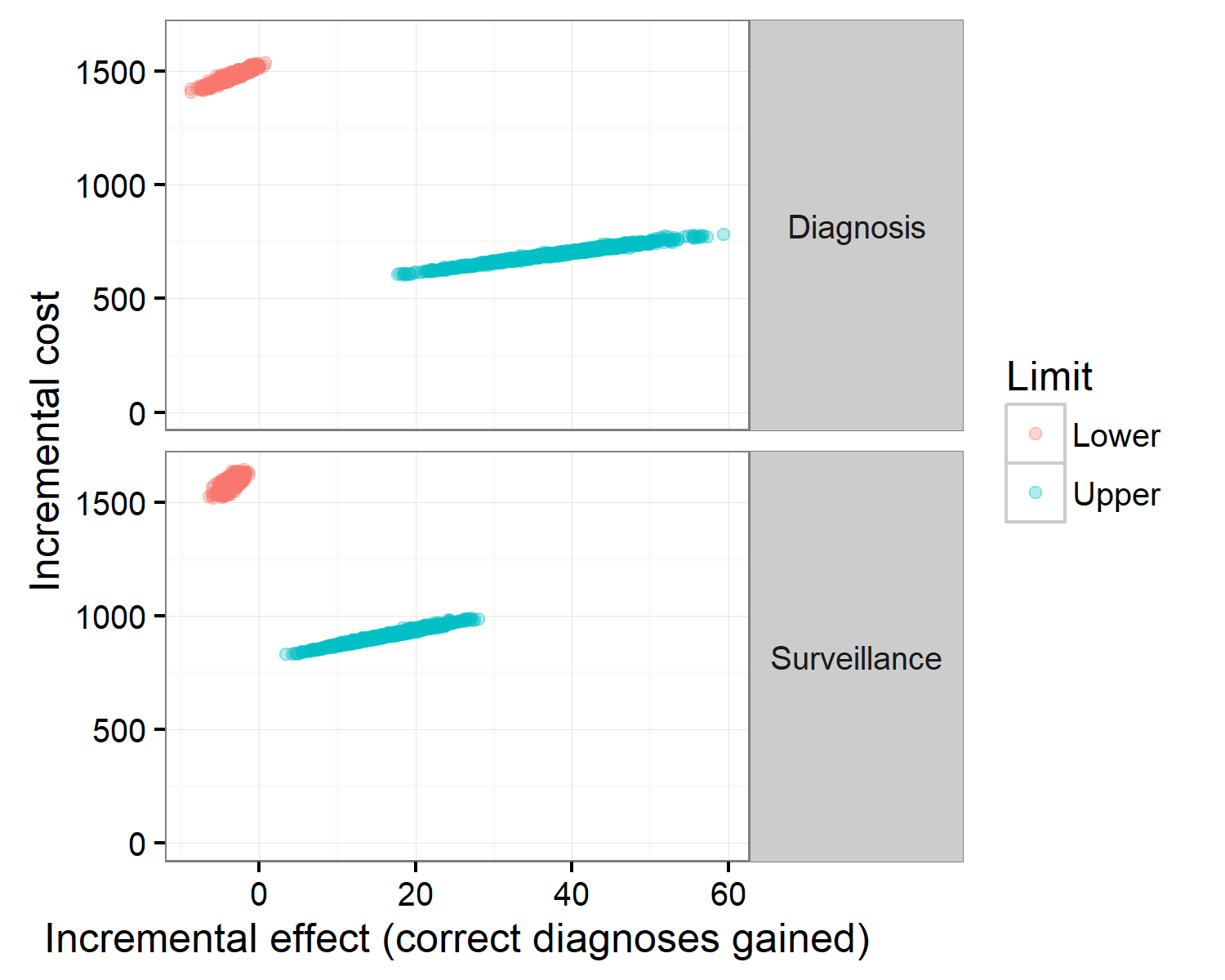

Supplement: S4 Fig — Dots represent the 1000 simulated values from the probabilistic sensitivity analysis; for individual diagnosis, the current/past infection prevalence ranges from 20% (lower limit) to 55% (upper limit), of which 20% (lower limit) or 75% (upper limit) are currently infected; for community screening, current/past infection prevalence is 15–45%, of which 5–50% are currently infected; cost and effects are expressed per 1000 people tested. (TIFF) [file pntd.0005985.s006.tiff]
